# Interpretation of Results of Studies Evaluating an Intervention Highlighted in Google Health News: A Cross-Sectional Study of News

**DOI:** 10.1371/journal.pone.0140889

**Published:** 2015-10-16

**Authors:** Romana Haneef, Clement Lazarus, Philippe Ravaud, Amélie Yavchitz, Isabelle Boutron

**Affiliations:** 1 INSERM, UMR 1153, Epidemiology and Biostatistics Sorbonne Paris Cité Center (CRESS), METHODS team, Paris, France; 2 Paris Descartes University, Sorbonne Paris Cité, Faculté de Médecine, Paris, France; 3 Centre d'Épidémiologie Clinique, AP-HP (Assistance Publique des Hôpitaux de Paris), Hôpital Hôtel Dieu, Paris, France; 4 French Cochrane Center, Paris, France; 5 Department of Epidemiology, Columbia University Mailman School of Public Health, New York, New York, United States of America; University of Geneva, SWITZERLAND

## Abstract

**Background:**

Mass media through the Internet is a powerful means of disseminating medical research. We aimed to determine whether and how the interpretation of research results is misrepresented by the use of “spin” in the health section of Google News. Spin was defined as specific way of reporting, from whatever motive (intentional or unintentional), to emphasize that the beneficial effect of the intervention is greater than that shown by the results.

**Methods:**

We conducted a cross-sectional study of news highlighted in the health section of US, UK and Canada editions of Google News between July 2013 and January 2014. We searched for news items for 3 days a week (i.e., Monday, Wednesday, and Friday) during 6 months and selected a sample of 130 news items reporting a scientific article evaluating the effect of an intervention on human health.

**Results:**

In total, 78% of the news did not provide a full reference or electronic link to the scientific article. We found at least one spin in 114 (88%) news items and 18 different types of spin in news. These spin were mainly related to misleading reporting (59%) such as not reporting adverse events that were reported in the scientific article (25%), misleading interpretation (69%) such as claiming a causal effect despite non-randomized study design (49%) and overgeneralization/misleading extrapolation (41%) of the results such as extrapolating a beneficial effect from an animal study to humans (21%). We also identified some new types of spin such as highlighting a single patient experience for the success of a new treatment instead of focusing on the group results.

**Conclusions:**

Interpretation of research results was frequently misrepresented in the health section of Google News. However, we do not know whether these spin were from the scientific articles themselves or added in the news.

## Background

Mass media through the Internet is an important and powerful means of disseminating and communicating medical research [1]. Especially, health news attracts large audiences and affects the behavior of healthcare providers and patients [2]. According to a report by Canadian Institute of Health Research, nearly 9 in 10 Canadians make decisions affecting their health as a direct result of media reports [3]. Such impacts may be beneficial, but high media coverage may have adverse effects. For example, a peak in media attention regarding group A streptococcal (GAS) disease and its testing in pediatric emergency departments was associated with an increase in the prescription of rapid tests for GAS despite no increase in the number of children presenting symptoms that might warrant such testing [2].

Undistorted dissemination of results of medical research is important to physicians, the scientific community and the public [4]. In theory, health news should be an accurate reflection of the research findings. Misrepresentation of study results to intentionally or unintentionally highlight that the beneficial effect of the intervention in terms of efficacy and safety is higher than that shown by the results is called “spin” [5]. Spin has been highlighted in the medical literature using various terms or synonyms such as distorted presentation [5, 6], misrepresentation [7, 8], exaggeration of research results [9–11], boasting [10], misleading or inadequate reporting [12, 13], biased interpretation [14], overinterpretation [6], or misinterpretation and inappropriate extrapolation [7]. This issue has been mainly addressed in case studies but also in some systematic assessments of cohorts of articles and press releases.

Previous studies have shown that spin is frequent in articles published in scientific journals, particularly in abstract conclusions [5], and that the presence of spin has an impact on readers’ interpretation [8]. Furthermore, spin in press releases and news items is related to the presence of spin in the abstract conclusions of published articles, and the findings of randomized controlled trials (RCTs) based on press releases and media coverage could be misinterpreted [7, 11].

Google News, which has one billion people a week using its news content, is one of the largest and most up-to-date online news services around the world [15]. Google News “watches” more than 4500 news sources worldwide. This service covers news articles appearing in the previous 30 days on various news websites. Google News aggregates content from more than 25,000 publishers. The health section of Google News includes online news citing new scientific research. However, to our knowledge, no critical assessment of the content of news items highlighted in the health section of Google News has been published.

We aimed to describe and assess the frequency of spin in news items reporting the results of studies evaluating an intervention that are highlighted in the health section of Google News.

## Methods

We conducted a cross-sectional study of news highlighted in the health section of Google News.

### Selection of health news referring to scientific articles

We systematically searched the health section of Google News (http://news.google.com/) for US, UK and Canada editions, 3 times a week (i.e., Monday, Wednesday and Friday) at the same time (14:00–17:00 Paris time) from July 19, 2013 to January 19, 2014. We arbitrarily selected these 3 country editions and working days. The duration of a given study highlighted in the health section of Google News varied from 30 min to 3 hours depending on the number of hits it received. Because of this “rapidly varying process” and lack of news archives of the front page, we systematically selected the news highlighted at a specific time.

In a first step, one researcher (RH) screened all the headlines of news appearing in the health section of Google News. News appearing in the health section has “real-time coverage” (i.e., all news reporting the same study at that time by different news sources but not highlighted on the front page). We included news that referred to a published study evaluating the effect of a treatment (pharmacological or non-pharmacological treatment) on human health regardless of study design. We also included any article published in any non-medical journal. We excluded news that reported 1) studies of correlation, screening, diagnostic, prognostic, case reports, guidelines and vaccine development; 2) highlighted the results of studies reported as an abstract or a poster presented in a scientific meeting or were unpublished; and 3) reported 2 or more scientific studies in one news item. If news dedicated to the same study appeared on the front page of more than one country edition by same or different news sources, only one of the news items was randomly selected.

In a second step, for previously selected news, the full text of the scientific articles was retrieved by using the reference of the article highlighted in the selected news or in “real-time coverage” of that news. If no reference was reported in the selected news, the name of the study author and the scientific journal that published the original study was searched in “real-time coverage” of the news. If the name of the scientific journal was mentioned, the author’s name was used to systematically search the current scientific journal issue or Google scholar, PubMed and Google. All retrieved articles were screened by 2 researchers (RH, CL).

### Classification of spin (misrepresentation of study results)

We defined “spin” as a specific way of reporting, from whatever motive (intentional or unintentional), to emphasize that the beneficial effect of the intervention is higher than that shown by the results [5].

We developed the classification of spin in 3 steps. First, we identified spin from a literature review on spin in published articles [5, 6, 12, 14, 16–27] and on reporting of scientific results in media and press releases [2, 4, 7, 9, 13, 28–38]. Second, we randomly selected a sample of 30 news items with or without spin to enrich our preliminary classification of spin. Third, the authors discussed the different types of spin retrieved and developed a classification of spin in 3 main categories: misleading reporting, misleading interpretation, and inadequate extrapolation.

#### Misleading reporting

Misleading reporting was defined as an incomplete or inadequate reporting of any important information in context of that research and that could be misleading for the reader. This category includes 1) not reporting adverse events; 2) misleading reporting of study design; 3) selective reporting of outcomes favoring the beneficial effect of the treatment (e.g., statistically significant results for efficacy outcomes or statistically non-significant results for safety outcomes); 4) linguistic spin (i.e., any word or expression emphasizing the beneficial effect of the treatment [10]); and 5) any other type of misleading reporting not classified under the above section.

#### Misleading interpretation

Misleading interpretation was defined as an interpretation of the study results in news not consistent with the results reported in the scientific articles and overestimating the beneficial effect of the treatment. This category includes 1) claiming a beneficial effect of the treatment despite statistically non-significant results; 2) claiming an equivalent effect of the treatment for statistically non-significant results; 3) claiming that the treatment is safe for statistically non-significant results despite lack of power; 4) claiming safety of the treatment despite adverse events reported in the scientific articles; 5) claiming a causal effect (i.e., implies a cause and effect relationship between the intervention being assessed and the outcome of interest [12]) despite non-randomized study design; 6) concluding a beneficial effect despite lack of a comparator; 7) focus on p-value instead of clinical importance; 8) interpretation of relative risk as absolute risk; and 9) any other type of misleading interpretation not classified under the above section.

#### Overgeneralization/misleading extrapolation

Overgeneralization/misleading extrapolation was defined as overgeneralization of study results in news to different populations, interventions or outcomes that were not assessed by the study. This category includes 1) extrapolation of animal study results to human application; 2) extrapolation of preliminary study results to clinical application; 3) extrapolating the effect of study outcomes to other outcomes for the disease; 4) extrapolation of the beneficial effect of the study intervention to a different intervention (e.g., broccoli, which contains sulphoraphane, was claimed as beneficial by health news, but the study evaluated the benefit of a sulphoraphane compound only); 5) extrapolation from the study participants to a larger or different population; 6) inappropriate implication for clinical or daily use (i.e., an improper recommendation or advice to use the intervention in clinical practice or daily use not supported by study results); and 7) any other types of extrapolation not classified under the above section.

All other spin that could not be classified with this scheme were systematically recorded and secondarily classified.

### Data extraction

Two researchers (RH, CL) with expertise in clinical epidemiology systematically read the abstract, methods and results sections of the scientific article and independently extracted data from the news using a preliminarily tested data extraction form. Two researchers (RH, IB) tested the form on a randomly selected sample of 10 news items by reading the referenced article and the content of the selected news items to extract specific information for spin. We evaluated the spin only in the health news. Discrepancies were resolved by discussion until consensus. If needed, a third researcher (IB) appraised the news and related article. The concordance between 2 reviewers for the assessment of spin is reported in S1 Table; the overall kappa coefficient was 0.65 [95% 0.48–0.82].

The following data were collected:

1. **General characteristics of health news:** we recorded the type of online news outlet (general news outlet dedicated to several domains including health such as BBC or health-specific news outlet dedicated to health only such as Medscape). We evaluated whether the following information were reported in the news: study population, study design, sample size, study limitations and funding source. We considered that the study design was reported in the news if it mentioned how the intervention was assigned to the study sample. We also assessed whether the news cited a full reference or an electronic link to the published article.
2. **General characteristics of published articles:** we recorded the journal type (i.e., specialized or general medical journal), study population (human and animal), study design (RCT, observational study, etc.), sample size, and funding source (non-profit, profit, both).
3. **Prevalence of spin in news**

We assessed the presence of spin in 1) headlines and 2) the text of the news, which may include quotations by study authors, experts or patients, when available in news. We identified the spin in these 2 sections of the news according to our classification in 3 main categories.

### Statistical analysis

We calculated frequencies and percentages (%) for qualitative variables. Data with quantitative variables are expressed with medians and inter-quartile range (IQR).

## Results

### Selection of health news

**Fig 1:**
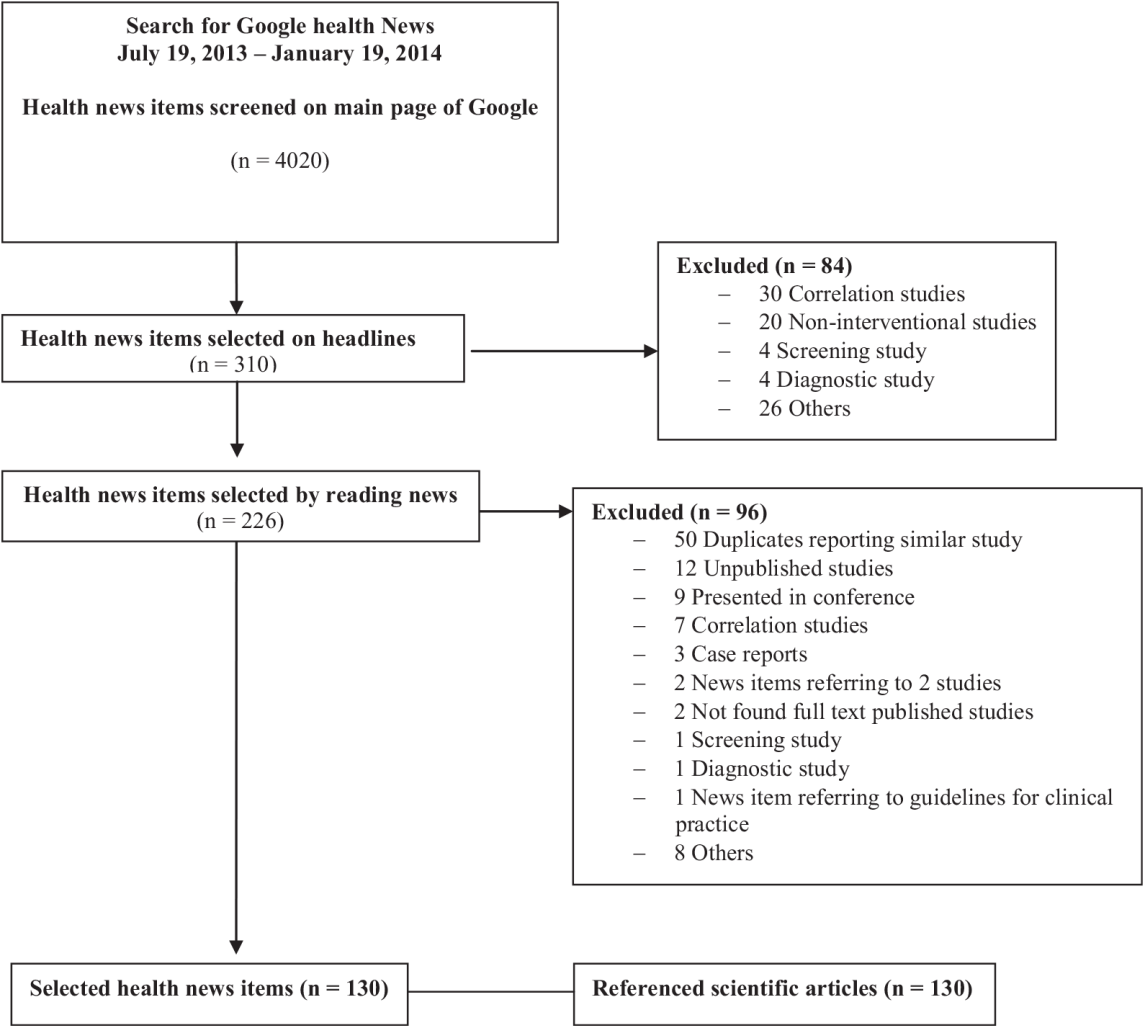
Flow diagram of selected Google health News with referenced scientific articles.

We screened 4,020 news items, of which 130 met our inclusion criteria and were included in this study are reported in Fig 1. The list of selected news items with referenced scientific articles is in S2 Table.

### General characteristics of health news

Overall, 98 (75%) of the news items were reported by a general news outlet (Table 1). The study population was reported in 90% of news items, the study design in 75% and the sample size in 68%. Some study limitations were reported in 44% of news items. Only 25% of items reported a funding source. In total, 78% of the news items did not report a full reference or electronic link to the published article.

**Table 1:** General characteristics of health news and scientific articles.

Category	
**Health news characteristics n = 130**	
**Type of online news outlet, n (%)**	
– General news outlet	75.4)
– *Medical news outlet*	32 (24.6)
**Reporting of**	
– Study population, n (%)	0.0)
– Study design, n (%)	75.4)
– Sample size, n (%)	67.7)
– Study limitations, n (%)	43.8)
– Funding source, n (%)	33 (25.4)
– Full reference or electronic link to the published article, n (%)	29 (22.3)
**Scientific article characteristics n = 130**	
**Type of journal, n (%)**	
– Specialized medical	58 (44.6)
– General medical	40.0)
– Life sciences	20 (15.4)
**Funding source, n (%)**	
– Non-profit	66.1)
– Profit	34 (24.1)
– Not reported	10 (7.7)
**Study population, n (%)**	
– Human	7.7)
– Animal	29 (22.3)
**Study design (if human study), n (%),**	**n = 101**
– Meta-analysis/ Systematic reviews	13.9)
– Randomized controlled trial	38.6)
– Cohort studies	32.7)
– Case–control	(5.1)
– Cross-sectional	(2.0)
– Before and after the intervention	8 (7.9)
**Sample size, median [IQR]** * (human studies)	634.5 [52–5208]

*[IQR], interquartile range

### General characteristics of scientific articles

Of the 130 scientific articles, 58 (45%) were published in specialized medical journals, 52 (40%) in general medical journals and 20 (15%) in life sciences journals (Table 1). The funding source was non-profit for 86 (66%). The study population was humans in 101 articles (78%) and animals in 29 (22%). Of the 101 articles of human studies, 14 (14%) were of meta-analysis and systematic reviews, 39 (39%) RCTs and 48 (48%) observational studies.

### Prevalence of spin in news headlines

We identified 58 (45%) news headlines with at least one spin (Table 2). Among news items reporting animal studies (n = 29), 48% (14/29) of headlines implied overgeneralization/misleading extrapolation from animals to humans. For example, in an animal study of rats evaluating a new treatment, the headline was “*Big breakthrough* *in cure for blindness*” with a picture of a human eye. This example contains 2 types of spin: first, the use of linguistic spin (i.e., “Big breakthrough”) and second, overgeneralization/misleading extrapolation from animals to humans. For news items reporting a non-randomized study design (n = 77), 22% (20/77) of headlines claimed a causal effect. For example, for a news item with the headline “*Vitamin D* *boosts* *strength of children*,” the study found only an association between maternal plasma 25 (OH) D statuses at 34 weeks’ gestation and children’s muscle strength at age 4 years in a non-randomized study design.

**Table 2:** Prevalence of spin in health news (n = 130).

Spin location	Spin, n/total news (%), n = 130
**Spin in headline, n (%)**	**58 (44.6)**
**Spin in text, n (%)**	
– No. of news reporting at least one spin	7.7)
– No. of spin, median; [IQR]; (min-max)	3 [1.0–4.0] (0–9.0)
**News with at least one misleading reporting** *	**(58.5)**
– Not reporting of adverse events‡	13/52 (25.0)
– Selective reporting of outcomes favoring statistically significant results	8.5)
– Misleading reporting of study design¥	2/48 (4.2)
– Linguistic spin or hype	63 (48.5)
**News with at least one misleading interpretation** *	**90 (69.2)**
– Claiming a beneficial effect of intervention despite statistically non-significant results	7 (5.4)
– Claiming the treatment is safe despite statistically non-significant results in treatment and comparison groups	(0.7)
– Claiming safety despite adverse events	4/52 (7.7)
– Claiming a causal effect despite non-randomized study design§	38/77 (49.3)
– Claiming a beneficial effect despite small sample size not reported ѱ	5/101 (5.0)
– Concluding a beneficial effect despite lack of comparator§	20/77 (25.9)
**News with at least one overgeneralization/misleading extrapolation** *	**40.8)**
– Results of animal study to human applicationǂ	6/29 (20.7)
– Preliminary study results to clinical application	12.3)
– Study outcomes to different outcomes	14.6)
– Study intervention to different interventions	10.0)
– Study participants to larger or different population	(6.9)
– Inappropriate implication for clinical/daily use	25 (19.2)
– Others	4 (3.1)
**Other spin**	**24 (18.5)**

*Several types are possible

‡ Only including human studies where adverse events were reported in scientific articles (n = 52)

¥ Applicable to observational studies (n = 48)

**§** Applicable to observational & animal studies (n = 77)

Ѱ Applicable to human studies (n = 101)

ǂ Applicable to animal studies (n = 29)

### Prevalence of spin in the text of news items

We identified 114 (88%) news items with at least one spin in the text (Table 2). The news items contained a median [IQR] of 3 [1.0–4.0] types of spin. We identified 18 types of spin in our sample of news (Table 3).

**Table 3:** Examples of 18 types of spin in health news.

Spin Categories	Spin type with *examples*	Explanation
**Misleading reporting**	Not reporting of adverse events‡: ***“Study uses stem cells to help treat drug-resistant TB”***	The study objective was to assess the safety of autologous mesenchymal stromal cell infusion as an adjunct treatment in patients with tuberculosis. In total, 217 adverse events were reported among all study subjects (i.e., 30) in a before-and-after study. However, the news did not report any adverse events.
	Selective reporting of study outcomes favoring statistically significant results: **“** ***Aspirin may reduce colon cancer in women”***	The study assessed the cancer incidence of breast, colon and lung cancer with low-dose aspirin. The study showed a statistically significant association between aspirin use and colon cancer (hazard ratio [HR] 0.80 [95% confidence interval (95% CI) 0.67–0.97]; p = 0.021) and a statistically non-significant association for breast cancer (HR 0.98 [95% CI 0.90–1.07]; p = 0.65) and lung cancer (HR 1.04 [95% CI 0.86–1.26]; p = 0.67). The news reported a significant association between only colon cancer and aspirin use.
	Misleading reporting of study design¥: “*The findings of****our trial****indicate that a good night’s sleep may be critical for maintain brain health”*.	The study design was not a trial but a before-and-after study of 15 healthy young study participants.
	Linguistic spin or hype: *“* ***Massive reduction*** *in side effects”*. *“* ***A radical drug*** *which lowers cholesterol by silencing a key gene could work just as well as statins but* ***without side-effects*** *and in just one dose*, *a study found*. *The medication has been hailed as a* ***Wonder drug***, *bringing down deaths from cardiac problems”*. *“* ***Big breakthrough*** *in cure for blindness”*.	Use of massive reduction, a radical drug, without side effects, wonder drug and big breakthrough are linguistic spins or hype.
**Misleading interpretation**	Claiming a beneficial effect of intervention despite statistically non-significant results: *“Participants in the study played games that were designed to train visual and spatial memory and quick decision making*. *Following the games*, ***older adults were able to stand up from being seated and walk faster than individuals who placed in a comparison group”***.	The study results did not show a statistically significant effect on gait (walk) speed (p = 0.124).
	Claiming the treatment is safe when results are statistically non-significant**: “** ***Gold injection did not alter urinary symptoms*** **”**.	The study reported similar dysfunctional symptoms in both groups in the study. No statistically test was performed to test the significance and data were provided in a figure.
	Claiming safety despite adverse events: *“* ***Our new approach using the patients’ own bone marrow stromal cells is safe*** *and could help overcome the body’s excessive inflammatory response*, *repair and regenerate inflammation-induced damage to lung tissue and lead to improved cure rates”*.	The study aimed to assess the safety of autologous mesenchymal stromal cell infusion as adjunct treatment in patients with tuberculosis. In total, 217 adverse events were reported among all subjects (i.e., 30) in a before-and-after study design.
	Claiming a causal effect despite non-randomized study design§: *“Breastfeeding* ***boosts*** *smarts as babies grow*, *the* ***longer babies are nursed*** **, *the*** ***greater their intelligence*** *”*.	The study assessed the association between breastfeeding duration and intelligence in a cohort design.
	Claiming a beneficial effect despite a small sample size not reported Ѱ: ***“Sleep protects your brain”***: *study*	The study assessed the effect of sleep intervention among 15 health young men in a before-and-after study design.
	Concluding a beneficial effect despite lack of comparator§: *“A new study has found that* ***watermelon juice can help post-exercise muscle soreness”***.	The study assessed the *in vitro* L-citrulline bioavailability from a synthetic standard or natural watermelon juice and determined the effect of a potential functional watermelon juice *in vivo* without a comparator group in a before-and-after study of 7 athletes.
**Overgeneralization/misleading extrapolation**	Results of animal study to human applicationǂ: *“Researchers have shown that contact lenses laced with medicines are an effective way* ***of*** ***treating glaucoma patients*** ***”***.	The rabbit study showed the effect only in rabbit eyes.
	Preliminary study results to clinical application: *“It could* ***treat phobias*** *and perhaps even* ***post-traumatic stress disorders”***	The study participants were healthy without any phobia and it was a very small sample of 15 subjects in a before-and-after study.
	Study outcomes to different outcomes: *1*. ***“*** ***Tomatoes may help fight breast cancer*** ***”*.** *2*. *“A radical drug which lowers cholesterol by silencing a key gene could work just as well as statins but without side-effects and in just one dose*, *a study found*. *The medication has been hailed as a Wonder drug*, ***bringing down deaths from cardiac problems”***.	1. The study examined the effects of diets rich in lycopene (tomato based) and isoflavone (soy based) on serum adipokine levels only. 2. The study did not assess effect of tomatoes based diet on decreasing the risk of breast cancer. The study did not assess the decrease in mortality with the ALN-PCS compound, which has not yet been developed as a drug.
	Study intervention to different interventions: *“* ***Broccoli*** *slows arthritis”*.	The study did not evaluate the use of broccoli but rather, sulphoraphane compound present in cruciferous vegetables, including broccoli, in a mouse study.
	Study participants to a larger or different population: *“The results of the trial-the first in humans-could offer hope to* ***one in five people who are resistant to statins*. *It could also be offered to patients who suffer ill-effects from the drugs*, *or those whose cholesterol remains high even after statins are prescribed”***.	The study participants were healthy with low-density lipoprotein cholesterol levels > 3.00 mmol/L and had received no lipid-lowering treatment in the 30 days before screening. The effect of the drug on participants with statin resistance was not evaluated in this study.
	Inappropriate implication for clinical/daily use: ***“Everyone should have at least 10–15 minutes of exposure to the sun every day to ensure that vitamin D levels are adequate”*.**	The rat study assessed dietary vitamin D deficiency leading to elevated tyrosine nitration in brain that may promote cognitive decline. The study did not assess the vitamin D level by exposure to sunlight.
	Other types of inappropriate extrapolations: ***“A new drug known as ALN-PCS***, *performed just as well*, *reducing cholesterol up to 57 per cent”*.	The study investigated the safety and efficacy of ALN-PCS, a small interfering RNA that is not yet developed as a drug. It was a randomized, single-blind, placebo-controlled, phase I trial.
**Others spin**	Highlighting a single patient experience for the success of a new treatment instead of focusing on the group results: ***“PROSTATE cancer patient Bob McGregor is living proof that a new treatment regime for the disease is as good as gold”***.	The study compared a 3-D conformal radiation therapy with and without image guidance using implanted fiducial markers in a cohort of 282 patients with prostate cancer with similar dysfunctional symptoms in both groups.

‡ Only including human studies where adverse events were reported in scientific articles (n = 52)

¥ Applicable to observational studies (n = 48)

**§** Applicable to observational & animal studies (n = 77)

Ѱ Applicable to human studies (n = 101)

ǂ Applicable to animal studies (n = 29)

Overall, 76 (59%) news items had at least one misleading reporting. One third of news items did not report adverse events, even though these were reported in the scientific articles. Use of linguistic spin or “hype” was identified in almost half of news items. For example, a news item stated *“A* *radical drug* *which lowers cholesterol by silencing a key gene […]*. *The medication has been hailed as a* *Wonder drug*, *bringing down deaths from cardiac problems”*.

A total of 90 (69%) news items had at least one misleading interpretation. Almost 49% of these items incorrectly claimed a causal effect of the intervention despite non-randomized study design (observational studies). For example, a news item reported that *“Daytime naps help* *improve* *learning in pre-school children by significantly* *enhancing* *their memories”*. Use of “improve” and “enhancing” implied a causal link between the intervention (daytime naps) and outcome (learning). This claim was inappropriate because the study was not randomized and the study design was a before-and-after study without a control group.

Finally, 53 (41%) news items had at least one overgeneralization/misleading extrapolation such as extrapolating a beneficial effect from an animal study to humans (21%). A news item reported that “*Researchers have shown that contact lenses […] are an effective way of treating* *glaucoma patients*”; the published study was on white rabbits. This item was reported with a photo of a woman holding a lens.

We also identified some new spin such as highlighting a single patient experience for the success of a new treatment. The interpretation should focus on group results. For example, to highlight the success of a new treatment for prostate cancer, the news item reported that “*PROSTATE cancer patient Bob McGregor is* *living proof* *that a new treatment regime for the disease is* *as good as gold*”. Other types of spin implied that the treatment is available but that it was at a very early stage of development; for example, one news item announced, “*Here is good news for cancer patients* *[…]*,”about a study performed on 3 mice, and the treatment will not available for current cancer patients.

#### Spin in quotations

We identified 115 (89%) news items reported with at least one quoted comment, 44% (51/115) with at least one example of spin. Of the 167 quoted comments reported, 59% (99/167) were by the study authors, 37% (62/167) experts and 4% (6/167) patients. Spin was identified in 43% (43/99) of quoted comments by authors, 19% (12/62) experts and 83% (5/6) patients. For example, in a study with statistically non-significant results, the author’s quote was *“To me it’s one of the* *best things* *that have happened in my medical practice*. *It’s rare to see something that works* *so dramatically*. *We didn’t realize it was going to produce such a* *massive reduction* *in side effects*. *It’s very solid step forward*. *It enables new technology to be used properly*. *It’s well on the way to becoming the norm”*.

**Fig 2:**
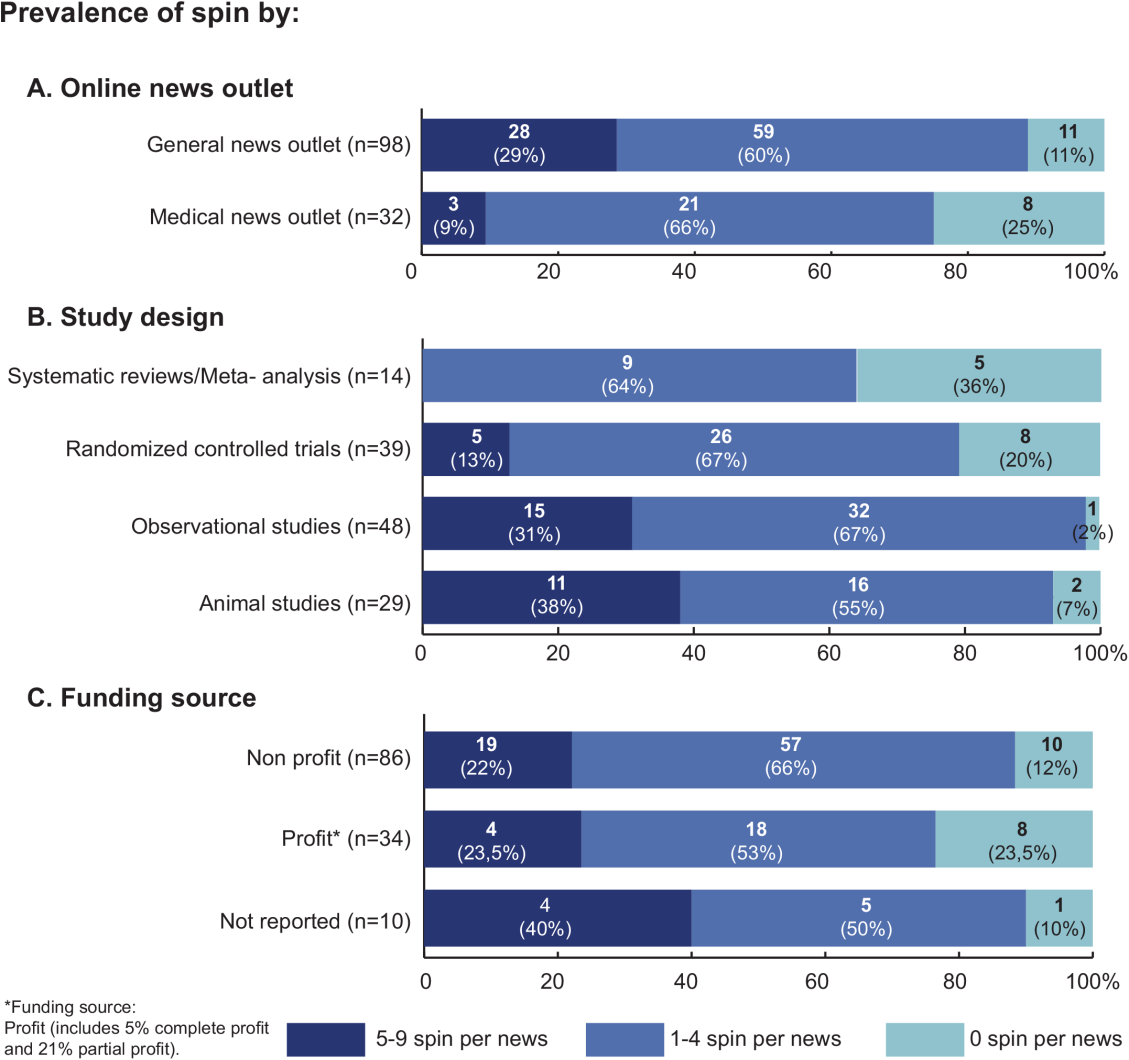
Prevalence of spin in online health news (n = 130).

The prevalence of spin by type of news outlet (panel A), study design (panel B) and funding source (panel C) are described in Fig 2.

## Discussion

Our evaluation of 130 news items that reported studies evaluating an intervention highlighted in the health section of Google News during a 6-month period showed a substantial misrepresentation of results. Among 130 news items, 88% contained at least one spin. These spin were mainly related to misleading reporting (59%), misleading interpretation (69%) and overgeneralization/misleading extrapolation (41%) of study results. However, we did not determine the source of the spin – scientific articles or added by journalists.

Research on spin in biomedical research is recent. Previous studies have focused on spin in RCTs [5, 7, 39, 40], diagnostic test accuracy studies [6, 41], non-randomized studies [14, 16, 42] and epidemiologic studies [43] and demonstrated a high prevalence of spin in published articles. A recently published RCT demonstrated that the presence of spin in abstract conclusions could have an impact on readers’ interpretation. [8] Other studies showed misrepresentation of research results in press releases [9, 28, 29, 31] and how it could have an impact on news [11] [31]. Furthermore, Yavchitz et al. showed that the presence of spin was associated with an overestimation of the beneficial effect of the treatment from research articles to press releases and the news [7]. Some studies have specifically assessed the contents of news and showed that the quality of reporting was poor, with important information missing [33] and a lack of reporting of the study limitations [44]. For example, in the United States [13] and in Canada [45], 53% and 68% of news stories, respectively, failed to mention the potential harms related to drug treatments for patients and failed to quantify the benefits.

To our knowledge, our study is the first to systematically assess the misrepresentation of research results highlighted in the health section of Google News, which has one billion users of its news content each week worldwide [15]. Our study provides a comprehensive evaluation and classification of spin in a highly disseminated sample of news reporting the results of scientific studies. We developed a classification of spin that could be applied equally to scientific research, press releases and news. Nevertheless, we cannot provide conclusions on the origin of the spin; indeed, the presence of spin in news could be related to the presence of spin in the published articles.

### Limitations

The first limitation is that the assessment of spin necessarily involves some subjectivity. Consequently, all reports were evaluated independently by 2 researchers. Second, we did not evaluate to what extent the spin was misleading for readers. The possible impact of spin on public perception about new treatments reported in health news should be studied. Third, our arbitrary selection of 3 country editions for Google News may limit the extrapolation of results to other country editions. Finally, we did not evaluate the origin of spin in news, whether it was due to journalists’ lack of scientific knowledge or from the published article.

### Implications

Misrepresentation of results can have serious consequences such as raising false hope among patients, distrust about new treatments, misguided choices that may put people’s health at risk or influence policy makers to adopt inadequate or harmful laws, regulations, or policies.

The implication of this study is to define strategic interventions to reduce the spin and the impact of spin on readers’ interpretation. These interventions could focus on researchers, journalists and the public. In fact, previous studies showed that spin in press releases and news items frequently came from the scientific articles [7, 11]. Consequently, to reduce the spin in news, the occurrence of spin should first be reduced in articles and then press releases. Second, we should train journalists to identify spin in scientific articles and avoid the dissemination of spin in the news. Finally, we should develop a users’ guide for the public to critically appraise news items and teach the public how to appraise health news critically. Some interesting initiatives [46, 47] such as “Behind the Headlines” [46], provide a critical analysis of health news stories.

Further research is recommended to assess the impact of spin on reader’s interpretation and public behaviour and which type of spin has high impact.

## Conclusions

In this sample of highly disseminated Google health news, the interpretation of research results was frequently misrepresented. However, we do not know whether these spin were from the scientific articles themselves or added in the news.

## Supporting Information

S1 Table Kappa coefficients for concordance on spin in Google health news items. (DOCX)

S2 Table List of the selected news items with referenced scientific articles and relevant news outlets. (XLSX)
