# Supplementary material for: Interpretation of Results of Studies Evaluating an Intervention Highlighted in Google Health News: A Cross-Sectional Study of News
Source: PLoS One. 2015 Oct 16;10(10):e0140889. doi: 10.1371/journal.pone.0140889 (PMC4608738; doi:10.1371/journal.pone.0140889)
Supplement: S1 Table — (DOCX) [file pone.0140889.s001.docx]

**S 1 Table: kappa coefficients for concordance on spin in Google health news items (N = 130)**

| **Variables** | **Agreement Percentage** | **kappa [95%CI]** |
| --- | --- | --- |
| News with at least one spin (Overall) | 89.2 | 0.653 [0.48 - 0.82] |
| Misleading Reporting | 77.7 | 0.552 [0.38 - 0.72] |
| Misleading interpretation | 92.2 | 0.365 [0.19 -0.53] |
| Overgeneralization/Misleading extrapolation | 81.5 | 0.567 [0.39 - 0.73] |
